# A Novel and Native Microcystin-Degrading Bacterium of *Sphingopyxis* sp. Isolated from Lake Taihu

**DOI:** 10.3390/ijerph14101187

**Published:** 2017-10-06

**Authors:** Juan Zhang, Qingqing Lu, Qin Ding, Lihong Yin, Yuepu Pu

**Affiliations:** Key Laboratory of Environmental Medicine Engineering of Ministry of Education, School of Public Health, Southeast University, Nanjing 210009, China; luqing_0215@163.com (Q.L.); dingqin@seu.edu.cn (Q.D.); lhyin@seu.edu.cn (L.Y.)

**Keywords:** microcystin, biodegradation, *Sphingopyxis* sp., Lake Taihu

## Abstract

A native, highly efficient microcystin-LR (MC-LR)-degrading bacterium named a7 was isolated from Lake Taihu and identified as *Sphingopyxis* sp. by 16S rDNA sequence analysis. The strain a7 could totally degrade MC-LR at a rate of 3.33 mg/(L·h), as detected by high-performance liquid chromatography (HPLC). The mlrA, mlrC, and mlrD genes were detected in the strain a7 by sequence analysis. Tetrapeptide and Adda—which are the middle metabolites of MC-LR—were analyzed via liquid chromatography time-of-flight mass spectrometry (LC-TOF-MS) during degradation. These metabolites were degraded completely, which suggested that the native *Sphingopyxis* sp. a7 was highly efficient in MC-LR degradation under bench conditions. Thus, strain a7 exhibited a significant potential application for bioremediation in water bodies contaminated by MC-LR produced by harmful cyanobacterial blooms.

## 1. Introduction

**Figure 1:**
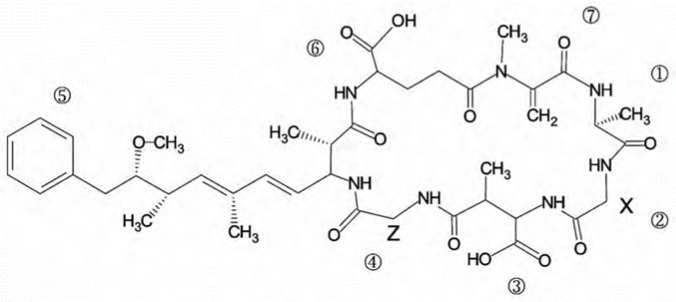
Chemical structure of microcystins (MCs). X and Z are variable l-amino acids [5].

Microcystins (MCs) are cyclic heptapeptides with seven amino acids, and are considered one of the most hazardous groups of toxins produced by harmful cyanobacterial blooms (HCBs) [1,2]. These toxins are cyclic heptapeptide hepatotoxins and tumor-promoting substances [3,4]. All MCs consist of the generalized structure cyclo-d-Ala^1^-X^2^-d-MeAsp^3^-Z^4^-Adda^5^-d-Glu^6^-Mdha^7^ (Figure 1) [5]. MC-LR, which has leucine (L) at position 2 and arginine (R) at position 4, is the most toxic among MCs [6]. Degradation products probably are linearized MC-LR, tetrapeptide, Adda, and other small molecules resulting from cleavage [7,8].

Various aquatic organisms, including molluscs, shrimp, and fish, accumulate MCs in their tissues [9,10,11]. Moreover, algal toxins in drinking water may exceed the standard amount during the bloom, which might increase the risk of cancers [11,12]. With the potential health risks, further studies should be conducted regarding the methods of MC removal.

MC toxins are nonvolatile, hydrophilic, and stable in sunlight over a wide temperature and pH range [13]. Consequently, they are difficult to remove by using the conventional water treatment of chlorine, chlorine dioxide, ozone, and permanganate [14,15]. The specific bacteria appearing along with cyanobacterial blooms can both lower and assimilate the algal toxins and their degradation products [16]. As one of the most important mechanisms for the removal of MCs from the natural environment, microbial degradation is an alternative strategy for physical and chemical water treatment programs [17,18].

Since the first isolation of the bacteria degrading MC in natural waters in 1994, a growing number of MC-degrading bacteria have been isolated in waters of Japan, China, and other parts of the world [19,20,21,22,23,24,25,26].

Lake Taihu, located in the delta of Yangtze River in Eastern China, is the third largest lake in China and offers a resource which supplies water to almost 2 million people for fishery, agriculture, industry, and household drinking. HCBs producing MCs have occurred regularly during the summer in a large part of Lake Taihu in the last three decades [17,22,27].

In previous research from our laboratory, the strain MC-LTH1 of *Bordetella* sp. and LTH2 of *Sarratiamarcesens* sp. can totally degrade MC-LR around the rates of 3.0 mg/(L·d) [22,28]. This study aimed to isolate a highly efficient native bacterium that can degrade MC-LR from Lake Taihu, and investigate the possible mechanism of MC degradation to bioremediate the local water environment.

## 2. Experimental Section

### 2.1. Collection, Domestication, Isolation, and Culture of MC-Degrading Bacteria

Sludge and water samples were collected from cyanobacteria-salvaged yards in the Guji district in Lake Taihu, China. The sludge (10 g, wet weight) was mixed with 90 mL of phosphate-buffered saline (PBS) by shaking at 30 °C for 30 min at 120 rpm. Subsequently, 0.5 mL of the supernatant was transferred to 4.5 mL of mineral salt medium (MSM) containing crude MC-LR (14.3 mg/L) and incubated with shaking at 30 °C [22]. MC-LR was detected during incubation every 5 days. The mineral salt medium (MSM, pH = 7.0) used for the bacterial isolation and culture contained (g/L) MgSO_4_·7H_2_O 1.0, KH_2_PO_4_ 0.5, K_2_HPO_4_ 4.0, NaCl 1.0, CaCl_2_ 0.02, FeSO_4_ 0.005, MnCl_2_·4H_2_O 0.005, ZnCl_2_ 0.005, and CuCl_2_ 0.0005 [18].

The samples that could degrade MC-LR were selected and treated as follows: MC-LR degradable bacteria taxa was serially diluted (1:10) with MSM containing crude MC-LR from 10^−2^ to 10^−10^. These diluted bacteria samples were cultured at 30 °C at 120 rpm for 5 days to degrade MC-LR completely. High-performance liquid chromatography (HPLC) was used to determine the MC-LR concentrations in different bacterial dilutions [28]. Bacterial colonies were selected from the highly efficient degradation sample with the highest dilution.

The selected bacteria (100 μL) were serially-diluted (1:10) with sterile PBS from 10^−2^ to 10^−10^. Afterward, 50 μL of diluted solution was spread out on the LB agar plates (2% agar) and cultured for 3 days at 30 °C. Every single colony with differing morphologies was selected and inoculated into MSM containing crude MCs to evaluate their degradation ability [29]. The colonies of MC-degrading bacteria were purified by plate-streaking technology on LB agar plates [30]. The bacterial culture and algal toxin liquid plate were crossed to determine the inbred strains. The inbred strains were transferred for the second verification.

### 2.2. Identification of 16S rDNA from MC-Degrading Bacteria

#### 2.2.1. Amplification of 16S rDNA

DNA extraction kit (Tiangen, Beijing, China) was used to extract the DNA of MC-degrading bacteria. The PCR reaction system was 50 μL, including 100 ng of template DNA, 1 × PCR Buffer (Mg^2+^ Free), 1.5 mM MgCl_2_, 0.2 mM dNTP mixture, 1.25 units of Taq DNA polymerase, and 400 nM of each of the following primers: 27F 5′-AGAGTTTGATCMTGGCTCAG-3′, 1492R 5′-TACGGYTACCTTGTTACGACTT-3′ [31]. The primers were synthesized by Shanghai Invitrogen.

The PCR condition included the initial denaturation at 98 °C for 5 min; 94 °C for 30 s, annealing for 30 s at 60 °C, 30 cycles of extension for 90 s at 72 °C; and a final elongation for 10 min at 72 °C. The PCR products were electrophoresed by agar gel with 2% agarose at 100 V for 40 min [31].

#### 2.2.2. Determination of 16S rDNA Sequence and Construction of Phylogenetic Tree

The PCR products of 16S rDNA were sent to the BGI Company (Shanghai, China) in 4 °C then sequenced. The sequencing results were submitted to the United States National Center for Biotechnology Information Website (NCBI) to obtain the corresponding registration number. Furthermore, nucleotide sequences were searched in GenBank for BLAST comparison, and the homology was analyzed. Sequence similarity search was conducted using the NCBI BLAST (https://blast.ncbi.nlm.nih.gov/Blast.cgi) network service [29]. Afterward, multiple sequence alignment and a phylogenetic tree were constructed using the software MEGA5 [32].

### 2.3. Cloning of Degrading Enzyme Genes from MC-Degrading Bacteria a7

mlrA, mlrB, mlrC, and mlrD specific primers were used to amplify the DNA of strain a7 according to the literature. The primer sequences are shown in Table 1. The PCR reaction system was 50 μL, which included 100 ng of template DNA, 1 × PCR buffer (Mg^2+^ Free), 1.5 mM MgCl_2_, 0.2 mM dNTP mixture, 0.4 mM of each primer, and 1.25 units of Taq DNA polymerase. The PCR condition included the initial denaturation at 95 °C for 5 min; 98 °C for 10 s, annealing for 30 s at 56 °C, 30 cycles of extension for 30 s at 72 °C; and a final elongation for 5 min at 72 °C and held at 4 °C. The PCR products were detected by the agarose gel electrophoresis and subsequently sequenced same with 16S rDNA.

The amino acid sequences of degrading enzyme genes were translated by the nucleotide sequences using the Sixpack program of EMBOSS (http://www.ebi.ac.uk/Tools/st/) [25]. The mlr genes were blasted by BLASTx program on the NCBI, and corresponding amino acid sequences were retrieved. Amino acid phylogenetic trees were constructed by MEGA5 software using the neighbor-joining algorithm based on distances calculated by Clustalw method [25].

### 2.4. Collection, Domestication, Isolation, and Culture of MC-Degrading Bacteria

The standard MC-LR was diluted with deionized water and MSM. The initial concentration of MC-LR was 20 mg/L. To investigate the degradation rate of standard MC-LR, the bacterial strain a7 was cultured on an orbital shaker at 120 rpm in MC-LR at 30 °C for 48 h in MSM containing crude MCs. The bacterial cells were centrifuged (5000× *g*, 15 min, 4 °C) and washed with PBS (Beyotime, Shanghai, China) twice. The bacterial strain a7 which was resuspended into MSM containing standard MC-LR was cultured at a constant condition (120 rpm, 30 °C) and analyzed by HPLC every 1 h [22].

MC-LR was measured by HPLC system (Agilent 1100, Santa Clara, CA, USA) with a Zorbax Extend C18 column (4.6 × 50 mm, 5 μm, Agilent, Santa Clara, CA, USA), which was kept at 40 °C. A mixture of methanol and 0.05% trifluoroacetic acid aqueous solution (53:47, *v*/*v*) was used as mobile phase. The flow rate was 1 mL/min, and the injection volume was 20 μL with 238 nm of variable wavelength detector [28].

The main degradation products of MC-LR were identified by a liquid chromatography time-of-flight mass spectrometry (LC-TOF-MS, Agilent 1200-6224, Santa Clara, CA,) in positive electrospray ionization (ESI) mode. A Zorbax Extend C18 column (2.1 × 50 mm, 1.8 μm, Agilent, Santa Clara, CA, USA) was used at 40 °C, and the injection volume was 2 μL. The mobile phase was a mixture of methanol and 0.1% formic acid aqueous solution (55:45, *v*/*v*) at a flow rate of 0.08 mL/min. The ESI-TOF-MS parameters were as follows: capillary voltage, 3500 V; gas temperature, 325 °C; flow of drying gas, 9 L/min; nebulizer pressure, 40 psi; and fragmentor voltage, 175 V. The flow rate was 0.1 mL/min [28].

## 3. Results

### 3.1. Isolation of MC-LR-Degrading Bacterium

According to the process of collection, domestication, isolation, and culture of MC-degrading bacteria, only one bacterium strain with high MC-degrading activities, named as a7, was obtained. In the selection of degrading bacteria, eight different colony types could survive in MC-LR media but could not degrade MC-LR.

### 3.2. Identification of MC-Degrading Bacterium by 16S rDNA Sequence Analysis

**Figure 2:**
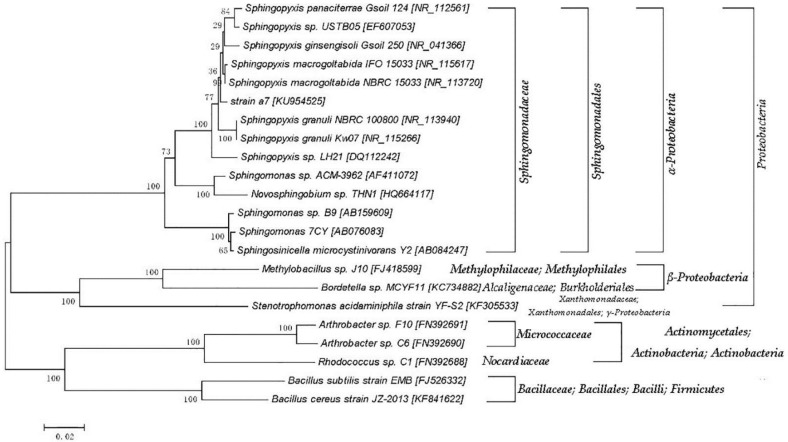
A phylogenetic tree based on the bacterial 16S rDNA sequence from the isolated strain a7 and the closely related species. The numbers at the nodes are the levels of bootstrap support (%) based on the neighbor-joining analyses of 1000 resampled datasets. The scale bar represents 0.02 nucleotide substitutions per position.

The PCR products of the 16S rDNA were sequenced by the BGI Company, and the accession number in the NCBI is KU954525. The phylogenetic tree was constructed with the homology of 97%. The nucleotide sequence of 16S rDNA from a7 was mostly similar to that of *Sphingopyxis macrogoltabida* strain NBRC 15033 (99% similarity, accession number: NR_113720.1). It suggested that strain a7 belongs to genus *Sphingopyxis*. The phylogenetic tree was constructed using a neighbor-joining analysis with 1000 bootstrap replication, as shown in Figure 2.

### 3.3. Cloning and Sequencing of the Key Genes of Degrading Enzyme from MC-Degrading Bacterium a7

**Figure 3:**
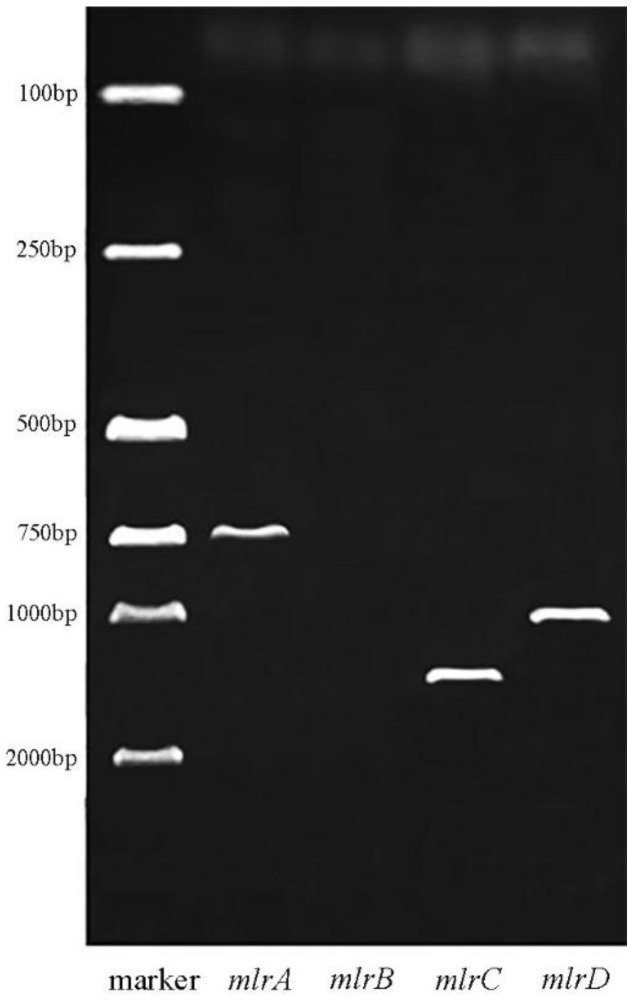
mlrA, mlrB, mlrC and mlrD PCR amplified products gel electrophoresis of strain a7.

**Figure 4:**
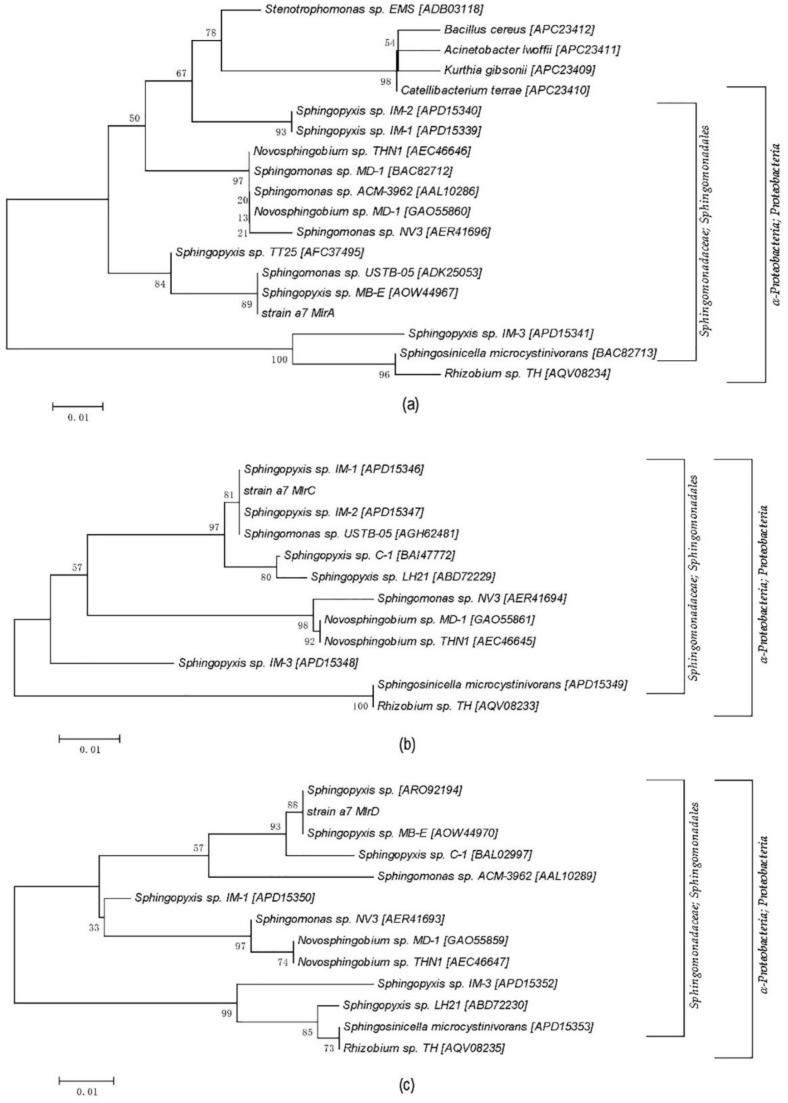
Amino acid sequences phylogenetic trees of mlrA, mlrC, and mlrD. The numbers at the nodes are the levels of bootstrap support (%) based on the neighbor-joining analyses of 1000 resampled datasets. The scale bar represents 0.01 estimated distance per position. (**a**) mlrA gene; (**b**) mlrC gene; (**c**) mlrD gene.

The mlrA, mlrB, mlrC, and mlrD specific primers were used for PCR amplification of a7, and the agarose gel electrophoresis of the PCR products is shown in Figure 3. Three bright DNA bands were observed after amplification, except for mlrB gene, and the fragment size was about 750, 1300, and 1000 bp. Thus, strain a7 contained the key enzyme gene mlrA, mlrC, and mlrD, which might be involved in the process of MC degradation. The PCR fragments were subsequently sequenced. The sequences of mlrA, mlrC, and mlrD from a7 were 99%, 100%, and 98% similar to the genes obtained from *Sphingomonas* sp. USTB-05, *Sphingomonas* sp. USTB-05, and *Sphingopyxis* sp. C-1, respectively. The amino acid phylogenetic trees of mlrA, mlrC, and mlrD genes were constructed based on the comparison with amino acid sequences of high-homology bacteria (MEGA5, Figure 4).

### 3.4. Biodegradation of MC-LR by Strain a7 and Analysis of Main Degradation Products

**Figure 5:**
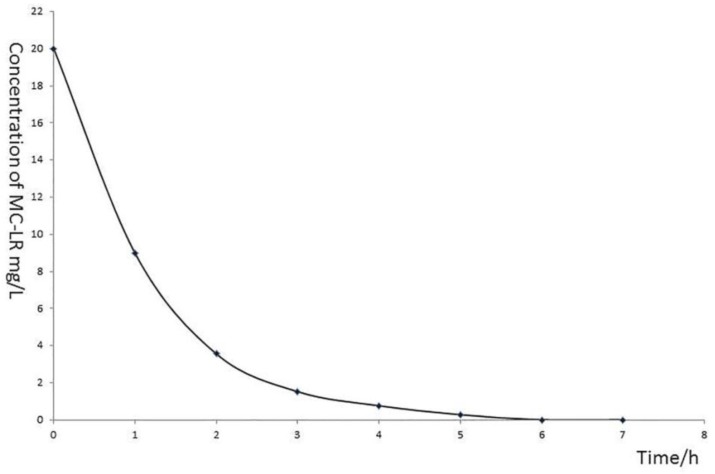
Degradation curve of standard MC-LR using strain a7.

**Figure 6:**
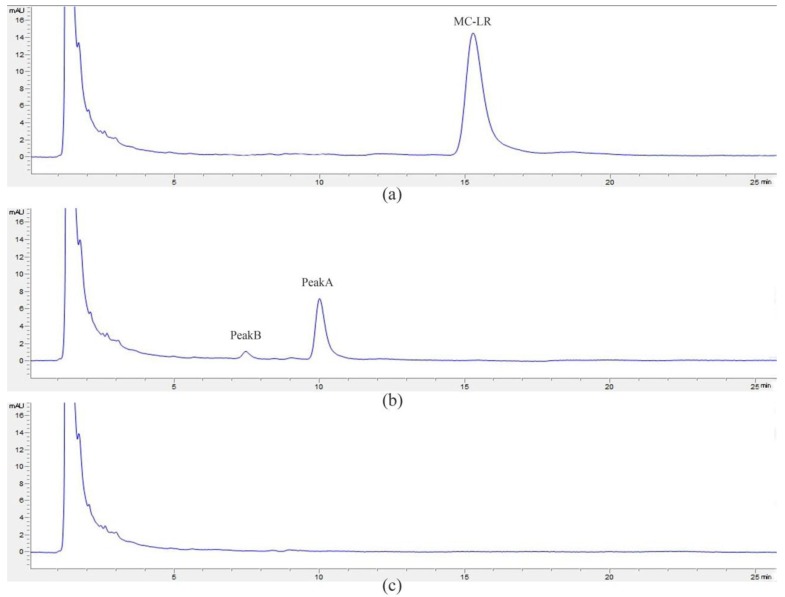
Degradation of standard microcystin LR using strain a7. High-performance liquid chromatography chromatogram at (**a**) 0, (**b**) 6 h, and (**c**) 12 h. Peaks A and B show two intermediate products of MC-LR.

About 20 mg/L MC-LR was completely degraded by strain a7 within 6 h, and the average degradation rate was 3.33 mg/(L·h) (Figure 5). MC-LR was degraded immediately without any lag phase, and more than 80% MC-LR was decomposed in the first two hours. Moreover, with the increase of time, degradation rate was progressively slower and eventually halted at 6h. Two major intermediate degradation products—namely, peaks A and B—were detected in 6 h using HPLC (Figure 6b). Strain a7 decomposed peaks A and B completely in 12 h (Figure 6c).

**Figure 7:**
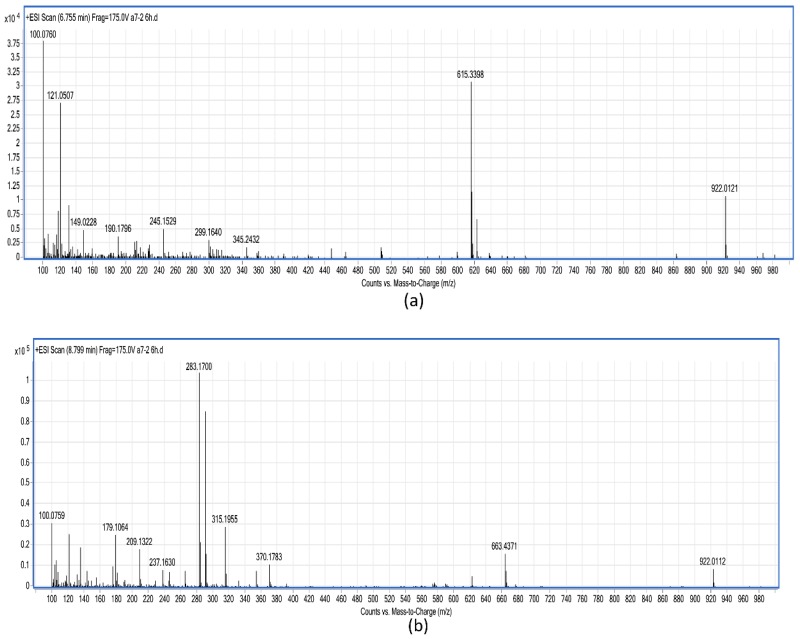
Electrospray ionization time-of-flight mass spectrometry (ESI-TOF-MS) spectrum of MC-LR degradation product. (**a**) MC-LR degradation product peak B with a molecular weight of 615.3398 ([M+H]^+^). (**b**) MC-LR degradation product peak A with a molecular weight of 283.1700 ([M+H−NH_3_−MeOH]^+^).

Peaks A and B in 6 h samples were further detected by LC-TOF-MS (Figure 7). The intermediate degradation product B exhibited a protonated molecular ion at *m*/*z* 615.3398 (C_32_H_46_N_4_O_8_), which was considered as tetrapeptide (Adda-Glu-Mdha-Ala) (Figure 7a). The intermediate product A exhibited several accompanying ions at *m*/*z* 315.1955 ([M+H−NH_3_]^+^) and 283.1700 ([M+H−NH_3_−MeOH]+), which were identified as Adda and its fragment, respectively (Figure 7b).

## 4. Discussion

In this study, an efficient and novel MC-degrading bacterium a7 was isolated from the bacterial communities of a lake. Moreover, 16srDNA of this a7 was characterized and identified as *Sphingopyxis. Sphingopyxis* sp. is a group of homogeneous microorganisms in the *α-Proteobacteria* class and has been isolated from nature samples before [16,19,26,35,36]. *Sphingopyxis* can tolerate the conditions of extreme poor nutrition, utilize various simple molecules, and decompose complex organic matter [26].

The degrading rate of MC by a7 was around 3.33 mg/(L·h). Compared with that of the previous studies, the degrading rate was six times greater than *Sphingomonas* sp. ACM-3962, 14 times of *Sphingomonas* sp. Y2, and hundredfolds of *Sphingopyxis* sp. LH21 which were designated as notable microorganisms with highly efficient of MC-degradation from natural waterbody [16,24,37,38]. The degradation rates varied from 0.06 μg/(L·d) to 101.52 mg/(L·d) in different research studies, probably caused by the different bacterial strains or other physical and chemical conditions utilized in the laboratory. Some studies have reported that MC-degrading bacteria had the most efficient degradation activity within 25–30 °C. The degrading enzymes were most probably inhibited when temperature was up 30 °C or below 25 °C [37,39,40]. So, in this study, the degrading rate was tested on 30 °C.

The discovery of bacteria that degrade MC-LR may indicate that these functional bacteria are prevalent in the natural environment [41]. Application of bacteria which are capable of decreasing microcystins dramatically during the outbreak of cyanobacterial-dominated harmful algal blooms is considered to be an important biological method toward diminishing the potential adverse effects of microcystins [28]. Table 2 shows the MC-degrading rates of bacteria isolated in individual research and different areas in the world.

**Figure 8:**
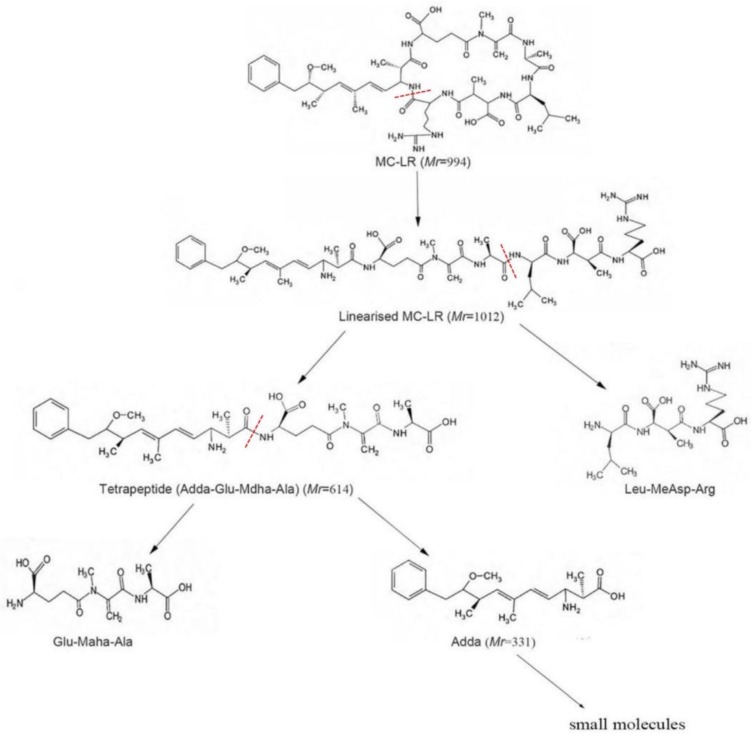
Possible degradation pathway of MC-LR by strain a7. The position of cleavage is marked with an arrow (↓).

The MC-degrading capability was attributed to mlrA, mlrB, mlrC, and mlrD genes in the bacteria reported by Kormas and et al. [6,7,25,37]. Based on the results of the presented study, microcystinase (MlrA) hydrolyzes cyclic MC-LR into a linear intermediate (Adda-Glu-Mdha-Ala-Leu-MeAsp-Arg) by undermining the peptide bond connecting Adda and arginine [25]. Further decomposition of linearized MC-LR by MlrB to generated a tetrapeptide (Adda-Glu-Mdha-Ala, 614, C_32_H_46_N_4_O_8_) [16]. MlrC exhibited a double-degrading activity, which was decomposing both linear MC-LR and the tetrapeptide into Adda (331, C_20_H_29_NO_3_) and other small molecular substances [7]. MlrD is predicted to transport MC-LR and its degradation products across the bacterial cell wall [37]. In our study, the ion of degradation product B presented a similar molecular weight (615.3398) with tetrapeptide. Therefore, degradation product B was determined as tetrapeptide. The protonated ion A (*m*/*z* 332.2208) was consistent with Adda. Ions at *m*/*z* 315.1955 (consistent with [adda+H-NH_3_]^+^) and 283.1700 (consistent with [adda+H−NH_3_−MeOH]^+^) were fragments of Adda. So, the degradation product A can be determined as Adda generally. Furthermore, mlrA, mlrC, and mlrD were detected in *Sphingopyxis* a7. Therefore, the possible degradation pathway of MC-LR might be shown in Figure 8 and MC-LR can be degraded by *Sphingopyxis* sp. a7 completely. To our knowledge, this study is the first report that MC-LR and Adda which is the main active structure of MC-LR can be decomposed simultaneously using single bacterial strain *Sphingopyxis* sp. [7,16,34]. MC-degrading and detoxication properties of strain a7 can potentially be applied for bioremediation in the harmful cyanobacterial blooms and contribute to improve water quality.

## 5. Conclusions

A native and highly efficient MC-LR-degrading bacterium was isolated from Lake Taihu and identified as *Sphingopyxis* sp., according to the 16S rDNA sequence. The MC-LR degrading rate was 3.33 mg/(L·h) at bench conditions. Strain a7 contained the key enzyme genes mlrA, mlrC, and mlrD, which might be involved in MC degradation. The degradation products included tetrapeptide and Adda, which were the metabolites of MC-LR catalyzed by microcystinases. This is the first report finding that MC-LR and Adda can be decomposed totally by *Sphingopyxis* sp.

Therefore, isolated *Sphingopyxis* sp. a7 may be used to solve the problem of MC-LR pollution. Further studies should apply a7 to an actual environmental bioremediation project.

## Figures and Tables

**Table 1:** Specific primer sequences.

Primers	Sequence	References
mlrA-Forward	5′-GACCCGATGTTCAAGATACT-3′	[33]
mlrA-Reverse	5′-CTCCTCCCACAAATCAGGAC-3′	
mlrB-Forward	5′-CTCGATGCGGTATTTGCTG-3′	[34]
mlrB-Reverse	5′-TCCAACGACCATCCCTTCTG-3′	
mlrC-Forward	5′-CGAAGGCGAAAGGTGCAAC-3′	[35]
mlrC-Reverse	5′-GAGCGCTTGTGATAGTGACG-3′	
mlrD-Forward	5′-GTTCCTCGGCGTAGCCT-3′	[24]
mlrD-Reverse	5′-GCGACGAAGATCGTTGCT-3′	

**Table 2:** Microcystin-degrading bacteria isolated in different areas and degradation rates.

Bacteria	Source	16S rRNA GenBank Accession Number	Degradable Microcystin Analogues	Degradation Rate μg/(L·d)	References
*α-Proteobacteria*					
*Sphingopyxis* sp. USTB-05	-	EF607053	MC-RR; MC-YR	101,520; 35,520	[35,36,42]
*Sphingopyxis* sp. LH21	Torrens Lake in South Australia	DQ112242	MC-LR; MC-LA	2.08; 2.50	[24]
*Sphingomonas* sp. CBA4	San Roque reservoir	AY920497	MC-RR	130	[43]
*Sphingomonas* sp. 7CY	Surface water sample of Lake Suwa	AB076083	MC-LR; MC-RR	1500; 1000	[44]
*Sphingomonas* sp. Y2	A hypertrophic lake	NR_040927/AB084247	MC-LR; MC-RR; MC-YR	5400; 13,000; -	[37]
*Sphingomonas* sp. ACM-3962	Surface water, Australia	AF411072	MC-LR; MC-RR	12,100 ^#^; 12,100 ^#^	[16,38]
*Sphingomonas* sp. B9	Tsukui lake	AB159609	MC-LR; MC-RR	25; 75	[45]
*Sphingomonas stygia*	Natural lakes	-	MC-LR; MC-RR; MC-YR	660; 1900; 1250	[15]
*Novosphingobium* sp. THN1	Lake Taihu	HQ664117	MC-LR	2700	[46]
*β-Proteobacteria*					
*Bordetella* sp. MCYF11	Lake Taihu	KC734882	MC-LR; MC-RR	7440;4080	[22]
*Methylobacillus* sp. J10	Lake Taihu	FJ4185	MC-LR; MC-RR	4940; 6350	[47]
*Burkholderia* sp. UPC-BI05	A South Brazilian coastal lagoon surface water	DQ459360	MC-LR	50	[48]
*γ-Proteobacteria*					
*Pseudomonas aeruginosa*. DMxS	Patos Lagoon estuary	HQ890467	MC-LR	0.06	[49]
*Stenotrophomonas acidaminiphila* YF-S2	Lake Taihu	KF305533	MC-LR; MC-RR	3000; 5600	[28]
*Escherichia coli*	-	-	MC-LR	5270	[50]
*Pseudomonas aeruginosa*	Reservoir surface water	-	MC-LR	2500	[51]
*Morganella morganii* C25216	Las Vegas Bay of Lake Mead	-	MC-LR	4.75	[42]
*Stenotrophomonas* sp. EMS	Lake Taihu	-	MC-LR; MC-RR	700; 1700	[30]
*Firmicutes*					
*Bacillus* sp. JZ-2013	Lake Chaohu	KF841622	MC-LR	1330	[52]
*Bacillus* sp. EMB	Lake Taihu	FJ526332	MC-LR; MC-RR	2150; 2990	[53]
*Lactobacillus rhamnosus* GG and LC-705	Probiotic strains	-	MC-LR	65 and 55	[54]
*Bacillus flexus* SSZ01	a Saudi eutrophic lake	-	MC-RR	2500	[55]
*Actinobacteria*					
*Microbacterium* sp. DC8	Lake Okeechobee	-	MC-LR	8.60	[56]
*Bifidobacterium longum* 46	Probiotic strains	-	MC-LR	70	[54]
*Rhodococcus* sp. C1,C3	Scottish water bodies	FN392688, FN392689	MC-LR	4,000	[57]
*Arthrobacter* sp. C6, F7, F10, R1, R1, R6 and R9	Scottish water bodies	FN392690, FN392692-FN392697	MC-LR	5000, 5000, 4000, 5000, 5000, 5000, and 5000	[57]
*Brevibacterium* sp. F10	Scottish water bodies	FN392691	MC-LR	5000	[57]

-: no detection /no mention. #: Degradation rate of this strain calculated by Eleuterio and Batista.

## References

[1] Dawson R.M. (1998). The toxicology of microcystins. Toxicon.

[2] Agha R., Cires S., Wormer L., Quesada A. (2013). Limited stability of microcystins in oligopeptide compositions of *Microcystis aeruginosa* (*Cyanobacteria*): Implications in the definition of chemotypes. Toxins.

[3] Falconer I.R. (1998). Quality and Treatment of Drinking Water II: The Handbook of Environmental Chemistry.

[4] Humpage A.R., Hardy S.J., Moore E.J., Froscio S.M., Falconer I.R. (2000). Microcystins (Cyanobacterial toxins) in drinking water enhance the growth of aberrant crypt foci in the mouse colon. J. Toxicol. Environ. Health A.

[5] Codd G., Bell S., Kaya K., Ward C., Beattie K., Metcalf J. (1999). Cyanobacterial toxins, exposure routes and human health. Eur. J. Phycol..

[6] Imanishi S., Kato H., Mizuno M., Tsuji K., Harada K. (2005). Bacterial degradation of Microcystins and Nodularin. Chem. Res. Toxicol..

[7] Shimizu K., Maseda H., Okano K., Kurashima T., Kawauchi Y., Xue Q., Utsumi M., Zhang Z., Sugiura N. (2012). Enzymatic pathway for biodegrading Microcystin LR in *Sphingopyxis* sp. C-1. J. Biosci. Bioeng..

[8] Hashimoto E.H., Kato H., Kawasaki Y., Nozawa Y., Tsuji K., Hirooka E.Y., Harada K. (2009). Further investigation of microbial degradation of microcystin using the advanced Marfey method. Chem. Res. Toxicol..

[9] Gurbuz F., Uzunmehmetoglu O.Y., Diler O., Metcalf J.S., Codd G.A. (2016). Occurrence of Microcystins in water, bloom, sediment and fish from a public water supply. Sci. Total Environ..

[10] Paerl H.W., Huisman J. (2009). Climate change: A catalyst for global expansion of harmful Cyanobacterial blooms. Environ. Microbiol. Rep..

[11] Yu S.Z., Tang Z.Y., Wu M.C., Xia S.S. (1989). Drinking water and primary liver cancer. Primary Liver Cancer.

[12] Ueno Y., Nagata S., Tsutsumi T., Hasegawa A., Watanabe M.F., Park H.D., Chen G.C., Chen G., Yu S.Z. (1996). Detection of Microcystins, a blue-green algal Hepatotoxin, in drinking water sampled in Haimen and Fusui, endemic areas of primary liver cancer in China, by highly sensitive immunoassay. Carcinogenesis.

[13] World Health Organization (2003). Cyanobacterial Toxins: Microcystin-LR in Drinking-Water. Background Document for Development of WHO Guidelines for Drinking-Water Quality.

[14] Lawton L.A., Robertson P. (1999). Physico-chemical treatment methods for the removal of Microcystins (Cyanobacterial Hepatotoxins) from potable waters. Chem. Soc. Rev..

[15] Saitou T., Sugiura N., Itayama T., Inamori Y., Matsumura M. (2003). Degradation characteristics of Microcystins by isolated bacteria from Lake Kasumigaura. J. Water Supply Res. Technol.–AQUA.

[16] Bourne D.G., Jones G.J., Blakeley R.L., Jones A., Negri A.P., Riddles P. (1996). Enzymatic pathway for the bacterial degradation of the Cyanobacterial cyclic peptide toxin Microcystin LR. Appl. Environ. Microbiol..

[17] Chen J., Hu L.B., Zhou W., Yan S.H., Yang J.D., Xue Y.F., Shi Z.Q. (2010). Degradation of Microcystin-LR and RR by a *Stenotrophomonas* sp. strain EMS isolated from Lake Taihu, China. Int. J. Mol. Sci..

[18] Gagala I., Mankiewicz-Boczek J. (2012). The natural degradation of Microcystins (Cyanobacterial Hepatotoxins) in fresh water—The future of modern treatment systems and water quality improvement. Pol. J. Environ. Stud..

[19] Jones G.J., Bourne D.G., Blakeley R.L., Doelle H. (1994). Degradation of the Cyanobacterial Hepatotoxin Microcystin by aquatic bacteria. Nat. Toxins.

[20] Okano K., Shimizu K., Kawauchi Y., Maseda H., Utsumi M., Zhang Z., Neilan B.A., Sugiura N. (2009). Characteristics of a Microcystin-degrading bacterium under Alkaline environmental conditions. J. Toxicol..

[21] Kazuya S., Kunihiro O., Hideaki M., Tomoaki I., Yukio K. Finding of Microcystin-Degrading Bacterium and Elucidation of Its Degradation Mechanism. https://www.pref.ibaraki.jp/soshiki/seikatsukankyo/kasumigauraesc/04_kenkyu/kaigi/docments/kosyou/13/2009wlc_SU.pdf.

[22] Yang F., Zhou Y., Sun R., Wei H., Li Y., Yin L., Pu Y. (2014). Biodegradation of microcystin-LR and-RR by a novel microcystin-degrading bacterium isolated from Lake Taihu. Biodegradation.

[23] Ho L., Hoefel D., Saint C.P., Newcombe G. (2007). Isolation and identification of a novel Microcystin-degrading bacterium from a biological sand filter. Water Res..

[24] Phujomjai Y., Somdee A., Somdee T. (2016). Biodegradation of microcystin [Dha(7)]MC-LR by a novel microcystin-degrading bacterium in an internal airlift loop bioreactor. Water Sci. Technol..

[25] Kormas K.A., Lymperopoulou D.S. (2013). Cyanobacterial Toxin Degrading Bacteria: Who Are They?. Biomed. Res. Int..

[26] Berg K.A., Lyra C., Sivonen K., Paulin L., Suomalainen S., Tuomi P., Rapala J. (2009). High diversity of cultivable heterotrophic bacteria in association with Cyanobacterial water blooms. ISME J..

[27] Zhang D., Xie P., Liu Y., Chen J., Liang G. (2007). Bioaccumulation of the hepatotoxic Microcystins in various organs of a freshwater snail from a subtropical Chinese lake, Taihu Lake, with dense toxic Microcystis blooms. Environ. Toxicol. Chem..

[28] Yang F., Zhou Y., Yin L., Zhu G., Liang G., Pu Y. (2014). Microcystin-degrading activity of an indigenous bacterial strain *Stenotrophomonas acidaminiphila* MC-LTH2 isolated from Lake Taihu. PLoS ONE.

[29] Li H., Ai H.N., Kang L., Sun X.F., He Q. (2016). Simultaneous Microcystis algicidal and Microcystin degrading capability by a single *Acinetobacter* bacterial strain. Environ. Sci. Technol..

[30] Somdee T., Thunders M., Ruck J., Lys I., Allison M., Page R. (2013). Degradation of [Dha(7)]MC-LR by a microcystin degrading bacterium isolated from Lake Rotoiti, New Zealand. ISRN Microbiol..

[31] Yang F., Wei H.Y., Li X.Q., Li Y.H., Li X.B., Yin L.H., Pu Y.P. (2013). Isolation and characterization of an algicidal bacterium indigenous to lake Taihu with a red pigment able to lyse *Microcystis aeruginosa*. Biomed. Environ. Sci..

[32] Tamura K., Peterson D., Peterson N., Stecher G., Nei M., Kumar S. (2011). MEGA5: Molecular evolutionary genetics analysis using maximum likelihood, evolutionary distance, and maximum parsimony methods. Mol. Biol. Evol..

[33] Saito T., Okano K., Park H.D., Itayama T., Inamori Y., Neilan B.A., Burns B.P., Sugiura N. (2003). Detection and sequencing of the microcystin LR-degrading gene, mlrA, from new bacteria isolated from Japanese lakes. FEMS Microbiol. Lett..

[34] Shimizu K., Maseda H., Okano K., Itayama T., Kawauchi Y., Chen R., Utsumi M., Zhang Z., Sugiura N. (2011). How Microcystin-degrading bacteria express Microcystin degradation activity. Lakes Reserv. Res. Manag..

[35] Xu H., Wang H., Xu Q., Lv L., Yin C., Liu X., Du H., Yan H. (2015). Pathway for biodegrading microcystin-YR by *Sphingopyxis* sp. USTB-05. PLoS ONE.

[36] Zhang M., Pan G., Yan H. (2010). Microbial biodegradation of Microcystin-RR by bacterium *Sphingopyxis* sp. USTB-05. J. Environ. Sci..

[37] Bourne D.G., Riddles P., Jones G.J., Smith W., Blakeley R.L. (2001). Characterisation of a gene cluster involved in bacterial degradation of the Cyanobacterial toxin Microcystin LR. Environ. Toxicol..

[38] Jones G.J., Orr P.T. (1994). Release and degradation of Microcystin following algicide treatment of a Microcystis-aeruginosa bloom in a recreational lake, as determined by HPLC and protein phosphatase inhibition assay. Water Res..

[39] Chen X.G., Yang X., Yang L.L., Xiao B.D., Wu X.Q., Wang J.T., Wan H.G. (2010). An effective pathway for the removal of Microcystin LR via anoxic biodegradation in lake sediment. Water Res..

[40] Ho L., Hoefel D., Palazot S., Sawade E., Newcombe G., Saint C.P., Brooks J.D. (2010). Investigations into the biodegradation of Microcystin-LR in wastewaters. J. Hazard. Mater..

[41] Eleuterio L., Batista J.R. (2010). Biodegradation studies and sequencing of Microcystin-LR degrading bacteria isolated from a drinking water biofilter and a fresh water lake. Toxicon.

[42] Yan H., Wang J., Chen J., Wei W., Wang H., Wang H. (2012). Characterization of the first step involved in enzymatic pathway for Microcystin-RR biodegraded by *Sphingopyxis* sp. USTB-05. Chemosphere.

[43] Valeria A.M., Ricardo E.J., Stephan P., Alberto W.D. (2006). Degradation of Microcystin-RR by *Sphingomonas* sp. CBA4 isolated from San Roque reservoir (Córdoba–Argentina). Biodegradation.

[44] Ishii H., Nishijima M., Abe T. (2004). Characterization of degradation process of Cyanobacterial Hepatotoxins by a gram-negative aerobic bacterium. Water Res..

[45] Tsuji K., Asakawa M., Anzai Y., Sumino T., Harada K. (2006). Degradation of Microcystins using immobilized microorganism isolated in an eutrophic lake. Chemosphere.

[46] Jiang Y., Shao J., Wu X., Xu Y., Li R. (2011). Active and silent members in the *mlr* gene cluster of a Microcystin-degrading bacterium isolated from Lake Taihu, China. FEMS Microbiol. Lett..

[47] Hu L.B., Yang J.D., Zhou W., Yin Y.F., Chen J., Shi Z.Q. (2009). Isolation of a *Methylobacillus* sp. that degrades microcystin toxins associated with cyanobacteria. New Biotechnol..

[48] Lemes G.F., Kersanach R., Da S., Pinto L., Dellagostin O.A., Yunes J.S., Matthiensen A. (2008). Biodegradation of microcystins by aquatic *Burkholderia* sp. from a South Brazilian coastal lagoon. Ecotoxicol. Environ. Saf..

[49] Lemes G., Kist L.W., Bogo M.R., Yunes J.S. (2015). Biodegradation of [D-Leu(1)]microcystin-LR by a bacterium isolated from sediment of Patos Lagoon estuary, Brazil. J. Venomanim. Toxins.

[50] Dziga D., Lisznianska M., Wladyka B. (2014). Bioreactor study employing bacteria with enhanced activity toward Cyanobacterial toxins Microcystins. Toxins.

[51] Takenaka S., Watanabe M.F. (1997). Microcystin LR degradation by *Pseudomonas aeruginosa* alkaline protease. Chemosphere.

[52] Zhang J., Shi H., Liu A., Cao Z., Hao J., Gong R. (2015). Identification of a new Microcystin-degrading bacterium isolated from lake Chaohu, China. Bull. Environ. Contam. Toxicol..

[53] Hu L., Zhang F., Liu C., Wang M. (2012). Biodegradation of Microcystins by *Bacillus* sp. strain EMB. Energy Procedia.

[54] Nybom S.K., Dziga D., Heikkilä J.E., Kull T.J., Salminen S.J., Meriluoto J.O. (2012). Characterization of Microcystin-LR removal process in the presence of probiotic bacteria. Toxicon.

[55] Alamri S.A. (2012). Biodegradation of Microcystin-RR by *Bacillus flexus* isolated from a Saudi freshwater lake. Saudi J. Biol. Sci..

[56] Ramani A., Rein K., Shetty K.G., Jayachandran K. (2012). Microbial degradation of Microcystin in Florida’s freshwaters. Biodegradation.

[57] Manage P.M., Edwards C., Singh B.K., Lawton L.A. (2009). Isolation and identification of novel microcystin-degrading bacteria. Appl. Environ. Microbiol..

